# Rank-statistics based enrichment-site prediction algorithm developed for chromatin immunoprecipitation on chip experiments

**DOI:** 10.1186/1471-2105-7-434

**Published:** 2006-10-05

**Authors:** Srinka Ghosh, Heather A Hirsch, Edward Sekinger, Kevin Struhl, Thomas R Gingeras

**Affiliations:** 1 Affymetrix Inc., Santa Clara, CA 95051, USA; 2 Dept. Biological Chemistry & Molecular Pharmacology, Harvard Medical School, Boston, MA 02115, USA; 3 Ambion Inc., 2130 Woodward, Austin, TX 78744-1832, USA

## Abstract

**Background:**

High density oligonucleotide tiling arrays are an effective and powerful platform for conducting unbiased genome-wide studies. The *ab initio *probe selection method employed in tiling arrays is unbiased, and thus ensures consistent sampling across coding and non-coding regions of the genome. Tiling arrays are increasingly used in chromatin immunoprecipitation (IP) experiments (ChIP on chip). ChIP on chip facilitates the generation of genome-wide maps of in-vivo interactions between DNA-associated proteins including transcription factors and DNA. Analysis of the hybridization of an immunoprecipitated sample to a tiling array facilitates the identification of ChIP-enriched segments of the genome. These enriched segments are putative targets of antibody assayable regulatory elements. The enrichment response is not ubiquitous across the genome. Typically 5 to 10% of tiled probes manifest some significant enrichment. Depending upon the factor being studied, this response can drop to less than 1%. The detection and assessment of significance for interactions that emanate from non-canonical and/or un-annotated regions of the genome is especially challenging. This is the motivation behind the proposed algorithm.

**Results:**

We have proposed a novel rank and replicate statistics-based methodology for identifying and ascribing statistical confidence to regions of ChIP-enrichment. The algorithm is optimized for identification of sites that manifest low levels of enrichment but are true positives, as validated by alternative biochemical experiments. Although the method is described here in the context of ChIP on chip experiments, it can be generalized to any treatment-control experimental design. The results of the algorithm show a high degree of concordance with independent biochemical validation methods. The sensitivity and specificity of the algorithm have been characterized via quantitative PCR and independent computational approaches.

**Conclusion:**

The algorithm ranks all enrichment sites based on their intra-replicate ranks and inter-replicate rank consistency. Following the ranking, the method allows segmentation of sites based on a *meta *p-value, a composite array signal enrichment criterion, or a composite of these two measures. The sensitivities obtained subsequent to the segmentation of data using a *meta *p-value of 10^-5^, an array signal enrichment of 0.2 and a composite of these two values are 88%, 87% and 95%, respectively.

## Background

Eukaryotic gene/transcript expression is controlled by a complex combination of ordered events [1-5] coordinated by various regulatory elements. The primary regulatory elements associated with a transcript are: promoters, enhancers, silencers and response elements. The promoter, a cis-acting element, is located upstream in close proximity to the transcript it controls. The enhancers and silencers (negative regulatory regions) can act over significant distances to regulate gene expression. The response elements are the recognition sites of certain transcription factors (TFs); a majority of these are located within 1 kB of the transcriptional start site. The interplay between transcriptional activators/repressors, histone modifiers, remodeling complexes and the basal transcription machinery has been a subject of active research, and several fundamental questions remain. For example, the location and characteristics of the target regions, where the transcriptional regulators are bound, are poorly understood. DNA sequence motifs, which are considered potential markers, are at times weak predictors of regulatory targets. While the promoters constitute the canonical binding regions, the study of the dynamics of transcriptional regulation remains incomplete without an understanding of non-canonical sites and a comprehensive catalog of all possible enrichment sites. (Throughout this publication the term *enrichment site *refers to a region of ChIP enrichment in the immunoprecipitated sample, with respect to a control or to genomic DNA. Specifically, it could refer to TF binding sites (TFBS), RNA polymerase II (RNA pol II) binding occupancy, chromatin or histone modification sites, among others.) Another example is transcriptional regulation in individual cell lines, the details of which are also poorly understood. Depending on the cell-line, it is possible that each individual gene or transcript requires a different sequence of events to stimulate transcription. An understanding of the encoding of regulatory information is critical for the comprehension and codification of the functional roles of the protein-coding and non-coding components. These inquiries have prompted the development of various biochemical methodologies [6-8] as well as computational frameworks and models [9-11].

Generating a comprehensive catalog of enrichment sites and mapping the connectivity that underlies the transcriptional regulatory network mandates an unbiased genome-wide mapping technology. High density tiling arrays [11-17] are suitable, as they provide unprecedented base pair (bp) coverage and probe sequences in an unbiased manner, in both gene-rich and gene-poor regions of the genome. Contiguous blocks of the genome are tiled subsequent to the elimination of interspersed repeats and low complexity DNA sequences [18]. The union of a classical chromatin immunoprecipitation assay [14,19,20] with genomic tiling arrays facilitates an unbiased study of transcription factor binding and chromatin modification *in vivo*. ChIP on chip [11,13,14,19-21] has enabled researchers to localize and characterize regulatory targets. Evidence of TF binding to non-canonical sites, such as those at the 3' ends of genes or internal to genes [11] has also been shown. This category of TFBS can have weak array signal and p-value enrichment profiles. In such cases, reproducibility across replicate experiments, and characterization of experimental noise, are critical to the assessment of true positives [22]. While biochemical validation is the litmus test for TFBS it is not feasible at a genome wide level, underscoring the need for robust computational models and methods, such as the one proposed.

## Methods

### ChIP assay

Chromatin immunoprecipitation is a technique that enables mapping the *in vivo *enrichment sites of specific proteins of interest. It employs formaldehyde treatment of cells to covalently crosslink proteins to the DNA with which they are associated. The proteins are then isolated by immuno-affinity, which under ideal circumstances also isolate the associated DNA fragments. DNA is then recovered and analyzed by standard polymerase chain reaction (PCR) analysis. A shortcoming of this assay, in its standard form, is that it enables the study of a few target DNA regions at best, and therefore requires some *a priori *knowledge of appropriate regions for analysis. ChIP on chip obviates this limitation, and is therefore particularly effective for studying the dynamics of transcription factor binding in a genome-wide manner.

### Tiling arrays – The Affymetrix platform

These tiling arrays employ short oligonucleotide probe-pairs, of length 25 bases (25 mers) to interrogate a specified genomic region. Each probe-pair includes a perfect match (PM) and a mismatch (MM). The MM sequence is identical to its corresponding PM sequence, except for the central (13^th^) base. The objective of pairing a PM with a MM is to adjust for optical background noise and non-specific hybridization. A variety of tiling arrays with different probe and feature resolution are used for genome-wide transcription regulation studies. The probe resolution defines the center to center distance between two adjacent probes, in genomic space. A 22 bp probe resolution for 25 mers implies a 3 bp overlap (on average) between 2 adjacent probes. Currently, the probe resolution of the arrays encompasses a range from 5 bp-35 bp with feature resolution at 5μ and 10μ.

### Multi-factorial ChIP on chip experimental design

A generalized ChIP on chip experimental design for the study of a single TF could have total information content distributed across N arrays with J probes-pairs per array. The design could also include multiple cell lines (C), time points (T), and replicates (R), where the replicates are potentially of two types: biological (B) and technical (E). In totality, the multi-factorial experiment encompasses M arrays, where *M = *2 × *C *× *N *× *T *× *B *× *E*. The multiplier, 2, is indicative of a two-sample experiment comprising a control (CO) and treatment (TR). The control is the pull-down of genomic DNA or a non-specific antibody, and the treatment is the chromatin immunoprecipitated sample.

### Preliminary data analysis

The following fundamental steps in tiling array data processing are applied across the entire ChIP on chip dataset comprising M arrays. The steps include:

i) Background subtraction: PM-MM;

ii) Data normalization; [23-25]: median scaling and quantile normalization;

iii) Estimation of signal expression, ChIP or signal enrichment (SE), and p-value distribution. These distributions are computed using the Wilcoxon signed rank test (for p-value) and its associated Hodges Lehmann(HL) estimator (for SE)[11,26,27]. These metrics are estimated for all tiled probes per array. The SE and p-value distributions constitute the inputs to the proposed algorithm.

ChIP on chip assays frequently suffer technical artifacts due to reduced antibody specificity, variable reaction efficiencies during cross-linking of the TF to the genomic DNA, fragmentation of the bound DNA, immunoprecipitation, amplification and sample hybridization [14]. These artifacts can introduce non-biological variations in the scanned arrays and must be minimized in order to enhance the accuracy of data comparison across multiple replicates. Theoretically this should improve signal to noise ratio (SNR) in the data, underscoring true biological differences across samples. Therefore, prior to the generation of the p-value and SE distributions, a linear median scaling and quantile normalization [24,25] are implemented. These steps operate on feature-level signal intensities. The median scaling operation regards all PM and MM probes on arrays as equal entities. It is a two-step process which includes:

i) Computation of a global chip median (GCM) across all arrays;

ii) Linear scaling of each feature on an array such that the chip median for a given array is equal to the GCM. (Eqn. 1–2).

Treatment and control feature intensities are quantile normalized separately, and only within biological replicates.

*GCM *= *median*((*median*(*PM*_1_...*PM*_*J*_,*MM*_1_...*MM*_*J*_))_1_...,(*median*(*PM*_1_...*PM*_*J*_,*MM*_1_...*MM*_*J*_))_*M*_) *where J*: *total # of probe – pairs on an array *    Eqn. 1

*GCM *= *median*(*PM*'_1_...*PM*'_J_,*MM*'_1_...*MM*'_J_)_*m *_= *median*(*α*_1_*PM*_1_...*α*_*J*_*PM*_*J*_,*β*_1_*MM*_1_...*β*_*J*_*MM*_*J*_)_*m *_*where *1 ≥ *m *≥ *M and scale factors*: *α*_*J*_,*β*_*J *_    Eqn. 2

The Wilcoxon signed rank test and its associated HL estimator require knowledge of genomic alignment. Subsequent to normalization the probe-pairs are mapped to the genome using an exact 25 mer alignment of the PM sequence, and a probe-pair specific expression-level (S_rj_) is estimated. S_rj _refers to feature intensity, and can be modeled in terms of probe affinity, abundance, and multiplicative/additive noise components (Eqn. 3). Estimation of ChIP-enrichment entails measurement of the relative abundance of a nucleic acid sequence in an immunoprecipitated sample, with respect to a control sample. S_*rj *_is computed as positive log (*p-log*) transformation, on a per-replicate basis, individually for treatment and control (Eqn. 4). Data truncation as in a *p-log *transform can be avoided via a generalized log (*g-log*) transformation (Eqn. 5).

*S*_*rj *_≈ *I*_*rj *_= *a*_*j*_*A*_*rj*_*η*_*rj *_+ *φ*_*rj *_*where a*: *probe affinity*; *A*: *abundance*; *η*: *multiplicative noise*; *φ*: *additive noise *    Eqn. 3

*S*_*rj *_= *p *log(*PM *- *MM*)_*jr *_= log_2 _(max((*PM *- *MM*)_*j*_,1))_*r *_*where *1 ≥ *j *≥ *J and *1 ≥ *r *≥ *R *    Eqn. 4

*S*_*rj *_= *g *log(*PM *- *MM*)_*jr *_    Eqn. 5

The null hypothesis for the Wilcoxon signed-rank test states that two mutually independent sets of observations derived from two different populations (TR and CO respectively), have the same probability distribution; the common distribution is not specified [26,27]. In a two sample problem, the hypothesis is described by a location-shift model. This states that the two populations are the same, except that one is shifted from the other by an amount Δ (Eqn. 6), referred to as the location-shift parameter. The alternative hypothesis would thus state that Δ is either greater or less than 0. In the context of ChIP enrichment the null hypothesis implies there is no shift in location as a consequence of treatment. Since the focus of inquiry in ChIP on chip experiments is positive enrichment in treatment over control, a one-sided, upper-tail test is performed to compute the Wilcoxon test statistic (Eqn. 7). The p-value, estimated per probe, is restated as pScore (Eqn. 8) – negative log_10 _transformed p-value. The HL estimator is given by Eqn. 9.

The statistical power of the test is derived from the use of all non-redundant probe permutations across all treatment and control sample-pairs, and encompassed within a sliding window (W). The window, *W *= 2 *x BW *+ 1, centered about the index probe-pair, is parameterized in terms of bandwidth (BW). BW is computed in units of base-pairs (not probes) and is a constant for a given analysis. It is initialized based on an estimated average chromatin fragment length in the ChIP assay. Based on gel analysis, this length is estimated at 500 bp. Inclusion of the enrichment pattern of probes neighboring the index probe, but constrained within W, strengthens the analysis and mitigates noise spikes that may arise when considering the behavior of a single probe (25 bp). As best practice, multiple window sizes should be tested computationally to optimize for sensitivity and specificity. Within a given window the presence of repeat-masked probes may cause the distribution of probes flanking the index probe to be asymmetric. The optimization of a window based on the density of flanking probes is not recommended, since even in a tiling array the interrogation of the genomic sequence is semi-periodic and noncontiguous.

Δ = *p *log(*PM *- *MM*)_*jrTR *_- *p *log(*PM *- *MM*)_*jrCO *_= *S*_*rjTR *_- *S*_*rjco *_    Eqn. 6

*H*_0 _: Δ = 0 *and H*_*A *_: Δ > 0 *at the α level of significance *    Eqn. 7

*pScore *= *σ*_*p *_= -10(log_10_(*pValue*))     Eqn. 8

Δ^
 MathType@MTEF@5@5@+=feaafiart1ev1aaatCvAUfKttLearuWrP9MDH5MBPbIqV92AaeXatLxBI9gBaebbnrfifHhDYfgasaacH8akY=wiFfYdH8Gipec8Eeeu0xXdbba9frFj0=OqFfea0dXdd9vqai=hGuQ8kuc9pgc9s8qqaq=dirpe0xb9q8qiLsFr0=vr0=vr0dc8meaabaqaciaacaGaaeqabaqabeGadaaakeaacuqHuoargaqcaaaa@2E22@_*HL *_= *SE*_*j *_= *median*(*p *log(*PM *- *MM*)_*irT *_- *p *log(*PM *- *MM*)_*jrC*_) *where *1 ≥ *i *≥ *N*; 1 ≥ *j *≥ *N*; *N *= *Total # of probe – pairs in W*; *j*: *index probe Eqn. 9*

Jeong *et al *have demonstrated, via spectral analysis in the chromosomes of *E. coli*, a spatial pattern of transcriptional activity [28]. The authors used the autocorrelation function (ACF) [28,29] to estimate the degree of transcriptional similarity of individual transcripts along a chromosome, as a function of intervening distance. The ACF was approximated as a decaying function with statistically significant regions corresponding to relatively short inter-transcript distances. Let us make the assumption that a putative binding site spans *n *contiguous probes, whose intensities in the control and treatment (ChIP) are denoted by C_1_...C_n _and T_1 _... T_n _respectively. If these *n *probes do not constitute a true binding site and are independent, then the underlying noise should be stochastic. This is not true for tiling array data, where auto-correlation among neighboring probes can confound estimation of the true underlying enrichment and its discrimination from noise. The Wilcoxon does not test for spatial auto-correlation, which can disguise itself as moderate p-values [26,27]. The simplest means to correct for autocorrelation is to establish a stringent (Wilcoxon) p-value threshold, to minimize false positives. Another approach is to determine the auto-correlation factor from probe variance at the putative site, and estimate statistical confidence by comparing observed enrichment to a normal distribution whose variance is modulated by the auto-correlation factor.

### Binary segmentation for detection of enrichment sites

Common parametric approaches for the generation of enrichment sites employ binary segmentation of signal enrichment and/or p-value distribution computed across the tiled probes [11]. A p-value-based threshold (τ_p_) is used for segmentation of positive probes (P_j _= 1, j: probe index, Eqn. 10). This is followed by the clustering of contiguous – in genomic space – positive probes with a maximum gap (*maxgap*) of 500 bp between two adjacent positive probes and a minimum probe run (*minrun*) of 25 bp. The resultant probe clusters are labeled as putative enrichment sites.

Positive probe: *P*_*j *_= 1, *pValue*_*j *_≤ *τ*_*p *_*or pScore*_*j *_≥ *σ*_*p *_    Eqn. 10

Non-Positive probe: *P*_*j *_= 0, *pValue*_*j *_> *τ*_*p *_*or pScore*_*j *_<*σ*_*p*_

Threshold estimation is the critical component of binary segmentation. The threshold can be derived from either the pScore or the SE distribution. For multi-replicate data, the threshold can be derived from a composite pScore (SE) distribution generated by aggregating a probe-wise pseudo-median [26] across replicates. Alternatively, it can be derived from any one of the replicates selected at random or from the replicate experiment with highest sensitivity. The threshold can be a fixed value applied across all replicates, or a replicate-specific distribution-based value estimated from the 99th percentile (a user tunable parameter) of the pScore (SE) distribution. (The 99^th ^percentile is selected because approximately five percent of tiled probes manifest IP enrichment). Each option introduces a particular bias to the analysis, as discussed in the *Results*.

### Non-parametric algorithm

The proposed rank-statistics-based enrichment site prediction algorithm (RSSPA) is a non-parametric procedure built upon the framework of rank and replicate statistics.

The elements of RSSPA are:

i) Seeding of sites based on binary segmentation of data

ii) Optimization of sites based on centrality, variance, error and enrichment distributions

iii) Final segmentation of sites based on a stringent signal enrichment threshold

iv) Localization of site boundaries

Multi-replicate data is segmented based on their p-value and/or array signal enrichment (SE) distributions and putative ChIP sites are generated. Initially, the sites are ranked on an intra-replicate basis. RSSPA then assigns an overall rank to all ChIP-enriched sites based on co-optimization of intra-replicate rank and inter-replicate rank consistency. Sites with superior intra-replicate rank and high inter-replicate rank consistency dominate the population of sites with a low false discovery rate (FDR). The crux of this multivariate algorithm is the optimization combining minimization of p-value-based covariates with maximization of signal enrichment The outcome of RSSPA is threefold – detection of enrichment sites, ranking of these sites based on intra-replicate rank and inter-replicate rank consistency, and further segmentation of the ranked list of sites based on a *meta *p-value and/or array signal enrichment metrics. The performance of the algorithm is affected more by the reproducibility than the absolute number of the replicate experiments, combined in this analysis.

#### Step 1 – Seeding of sites based on binary segmentation of data

ChIP-enrichment emanating from true regulatory targets in the genome should be significantly higher than underlying noise. In this case a simple binary segmentation based on a moderate SNR threshold should detect these sites. Effective binary segmentation however requires accurate estimation of noise and absolute ChIP-enrichment profiles. Accurate noise estimation requires de-convolution of probe-level, array-level and assay-level effects – a more complex task in whole genome tiling arrays, where probes are not aggressively filtered to eliminate cross-hybridization effects. ChlP-enrichment is a measurement of the relative abundance of a specific nucleic acid sequence in an immunoprecipitated sample, and in the genomic DNA or non-specific control. Accurate estimation of this enrichment is highly dependant on quantification of the probe affinities and additive and multiplicative noise components in the experiment.

RSSPA does not incorporate estimation of absolute ChlP-enrichment profiles. To account for variance in sensitivity across replicates, RSSPA incorporates a ranked significance of enrichment. It also does not estimate the underlying noise, but assumes the noise profile of a given fragment of DNA remains approximately constant across all replicates. The cumulative probe level effect and the antibody specificity for a given fragment are assumed constant across replicates; the potential sources of variable noise are from fragmentation, amplification and array hybridization. The impact of the variable noise (variable across replicates) is mitigated via the optimization of the variance based covariate in the model. The site-level noise invariance across replicates assumes that the contribution of stochastic noise is low and does not perturb the overall prediction model (demonstrated in simulation results). Finally, the approach does not explicitly compute the auto-correlation effects, but mitigates false positives by co-optimization of p-Value and SE based metrics in the assignment of overall site rankings.

The initial stage of the algorithm comprises binary segmentation by application of a low pScore (SE) threshold, per replicate, yielding a minimum SNR of 1.1 (a user tunable parameter). pScore (p) and SE(s) thresholds, yielding a SNR of ~1.1:

i) Fixed thresholds: (a) σ_p _≥ 20; (b) τ_s _≥ ln(2) = 0.693

ii) Distributional thresholds: (a) σ_p _≥ 25^th ^percentile; (b) τ_s _≥ 25^th ^percentile.

This step obtains a set of the maximal number of *seed *intervals or sites, per replicate, at albeit a high false positive rate (FPR). In experiments with high noise floors, applying only a pScore threshold does have a tendency to result in over-segmentation of the data due to spatial auto-correlation. This can be mitigated by applying a combined pScore-SE threshold. The thresholding coupled with a maxgap and minrun of 500 and 25 bp are used to cluster neighboring positive probes into seed sites (Eqn. 11–Eqn. 12). By considering the pScore and SE distribution over all probes comprising a given site, a κ-trimmed mean (TrMean_κ_) summary estimate of the respective distributions is generated per seed site and replicate, for optimal κ = 0.20(Eqn. 13–Eqn. 14). Since seeding of sites based on p-value is more prone to false positives, the results have been discussed for this more nuanced approach. To distinguish between the two approaches, seed sites initialized via SE and p-value are labeled via α and β respectively; these labels are mentioned explicitly throughout the methods section but are omitted in the results section, for simplicity.

*α*_*r *_= *SeedSites*_*sig,r *_= *ℑ *(*τ*_*sig *_,*maxgap*, *minrun*, *TrMean*_*κ*_) *r *: *replicate*; 1 ≥ *r *≥ *R *    Eqn. 11

*β*_*r *_= *SeedSites*_*p-value*,*r *_= *ℑ*(*τ*_*pScore*_, *maxgap*, *minrun*, *TrMean*_*κ*_)     Eqn. 12

*SETM*_*s *_= (*TrMean*_*κ *_(*SE*)_*r*_)_*s *_    Eqn. 13

*PVTM*_*s *_= (*TrMean*_*κ *_(*p – value*)_*r*_)_*s *_    Eqn. 14

#### Step 2 – Optimization of sites based on centrality, variance and error distributions

In this stage, the seed site distributions are refined and statistical significance is assigned to each site. The site prediction model tests the null hypothesis that rank ordering of true targets across replicates is random. The rationale for this hypothesis is: when multiple biological replicates, which are aliquots of a population of cells (derived from a specific cell-line) are treated under equivalent experimental conditions, independent stretches of DNA which constitute targets of transcriptional regulation have a high probability of preserving the rank ordering of their signal enrichment and significance across replicates, while simultaneously manifesting a variance in enrichment [30]. In order to test the hypothesis the pScore and SE based covariates discussed below are computed across all seed sites and replicates.

Mechanistically, subsequent to binary segmentation sites are ranked individually in each replicate in descending order of magnitude (Eqn.15). These rankings are accompanied by a site-level *meta *p-value and composite SE as aggregated across replicates.  *ρ*_*α,r *_= *Rank*(*α*_*r*_, *order *= 0) *ρ*_*β,r *_= *Rank*(*β*_*r*_, *order *= 0)     (Eqn. 15) RSSPA follows a multivariate approach in which overall site rankings are assigned based on co-optimization of individual rank of sites within replicates and rank consistency and significance across replicates. The following three distributions computed based on the ranked pScore (β) across all seed sites and replicates constitute the covariates of analysis:

i) **μ**_**β **_is the centrality measure in the model. It is an average ranked pScore per site as aggregated across all replicates (Eqn. 16).

ii) **SAD**_**β **_is the variance measure in the model. It is the non-redundant sum of absolute pair-wise rank differences for a site across all replicate-pairs (Eqn. 17).

iii) **ε**_**β **_is the error measure in the model. It is the reciprocal of the *meta *p-value generated per site; the *meta *p-value is computed via the Fischer *χ*^2 ^transform of p-values across replicate datasets (Eqn. 18).

*μ*_*β,s *_= *average*(ρβ1
 MathType@MTEF@5@5@+=feaafiart1ev1aaatCvAUfKttLearuWrP9MDH5MBPbIqV92AaeXatLxBI9gBaebbnrfifHhDYfgasaacH8akY=wiFfYdH8Gipec8Eeeu0xXdbba9frFj0=OqFfea0dXdd9vqai=hGuQ8kuc9pgc9s8qqaq=dirpe0xb9q8qiLsFr0=vr0=vr0dc8meaabaqaciaacaGaaeqabaqabeGadaaakeaaiiGacqWFbpGCdaWgaaWcbaGae8NSdi2aaSbaaWqaaiabigdaXaqabaaaleqaaaaa@3163@ ... ρβr
 MathType@MTEF@5@5@+=feaafiart1ev1aaatCvAUfKttLearuWrP9MDH5MBPbIqV92AaeXatLxBI9gBaebbnrfifHhDYfgasaacH8akY=wiFfYdH8Gipec8Eeeu0xXdbba9frFj0=OqFfea0dXdd9vqai=hGuQ8kuc9pgc9s8qqaq=dirpe0xb9q8qiLsFr0=vr0=vr0dc8meaabaqaciaacaGaaeqabaqabeGadaaakeaaiiGacqWFbpGCdaWgaaWcbaGae8NSdi2aaSbaaWqaaiabdkhaYbqabaaaleqaaaaa@31E0@)_*s *_*s *: *site*; 1 ≥ *s *≥ *S*; *r *: *replicate*; 1 ≥ *r *≥ *R *    Eqn. 16

SADβ,s=12∑m,n=1r|ρβm−ρβn|sm,n∈r; m≠n     Eqn.17
 MathType@MTEF@5@5@+=feaafiart1ev1aaatCvAUfKttLearuWrP9MDH5MBPbIqV92AaeXatLxBI9gBaebbnrfifHhDYfgasaacH8akY=wiFfYdH8Gipec8Eeeu0xXdbba9frFj0=OqFfea0dXdd9vqai=hGuQ8kuc9pgc9s8qqaq=dirpe0xb9q8qiLsFr0=vr0=vr0dc8meaabaqaciaacaGaaeqabaqabeGadaaakeaafaqabeqacaaabaGaem4uamLaemyqaeKaemiraq0aaSbaaSqaaGGaciab=j7aIjabcYcaSiabdohaZbqabaGccqGH9aqpdaWcaaqaaiabigdaXaqaaiabikdaYaaadaaeWbqaamaaemaabaGae8xWdi3aaSbaaSqaaiab=j7aInaaBaaameaacqWGTbqBaeqaaaWcbeaakiabgkHiTiab=f8aYnaaBaaaleaacqWFYoGydaWgaaadbaGaemOBa4gabeaaaSqabaaakiaawEa7caGLiWoadaWgaaWcbaGaem4CamhabeaaaeaacqWGTbqBcqGGSaalcqWGUbGBcqGH9aqpcqaIXaqmaeaacqWGYbGCa0GaeyyeIuoaaOqaaiabd2gaTjabcYcaSiabd6gaUjabgIGiolabdkhaYjabcUda7iabbccaGiabd2gaTjabgcMi5kabd6gaUbaacaWLjaGaaCzcaiabbweafjabbghaXjabb6gaUjabc6caUiabigdaXiabiEda3aaa@654F@

χs2(df=2r)=−2∑r=1R(ln⁡(pValuer))s where, εβ,s∝1−10×log⁡(χs2,10)     Eqn.18
 MathType@MTEF@5@5@+=feaafiart1ev1aaatCvAUfKttLearuWrP9MDH5MBPbIqV92AaeXatLxBI9gBaebbnrfifHhDYfgasaacH8akY=wiFfYdH8Gipec8Eeeu0xXdbba9frFj0=OqFfea0dXdd9vqai=hGuQ8kuc9pgc9s8qqaq=dirpe0xb9q8qiLsFr0=vr0=vr0dc8meaabaqaciaacaGaaeqabaqabeGadaaakeaaiiGacqWFhpWydaqhaaWcbaGaem4CamhabaGaeGOmaidaaOGaeiikaGIaemizaqMaemOzayMaeyypa0JaeGOmaiJaemOCaiNaeiykaKIaeyypa0JaeyOeI0IaeGOmaiZaaabCaeaacqGGOaakcyGGSbaBcqGGUbGBcqGGOaakcqWGWbaCcqWGwbGvcqWGHbqycqWGSbaBcqWG1bqDcqWGLbqzdaWgaaWcbaGaemOCaihabeaakiabcMcaPiabcMcaPmaaBaaaleaacqWGZbWCaeqaaOGaeeiiaacaleaacqWGYbGCcqGH9aqpcqaIXaqmaeaacqWGsbGua0GaeyyeIuoakiabbEha3jabbIgaOjabbwgaLjabbkhaYjabbwgaLjabcYcaSiabbccaGiab=v7aLnaaBaaaleaacqWFYoGycqGGSaalcqWGZbWCaeqaaOGaeyyhIu7aaSaaaeaacqaIXaqmaeaacqGHsislcqaIXaqmcqaIWaamcqGHxdaTcyGGSbaBcqGGVbWBcqGGNbWzcqGGOaakcqWFhpWydaqhaaWcbaGaem4CamhabaGaeGOmaidaaOGaeiilaWIaeGymaeJaeGimaaJaeiykaKcaaiaaxMaacaWLjaGaeeyrauKaeeyCaeNaeeOBa4MaeiOla4IaeGymaeJaeGioaGdaaa@7F39@

**Figure 1:**
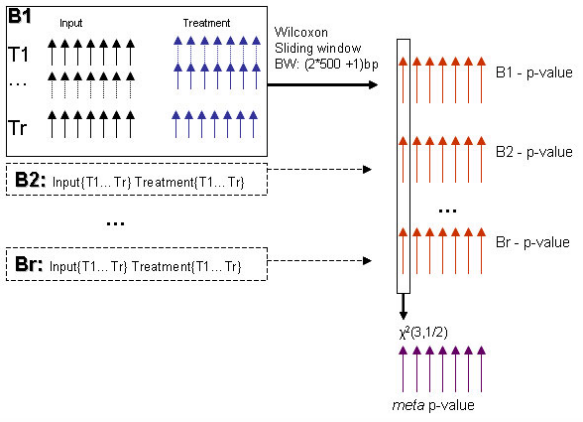
Schematic demonstrating the computation of a *χ*^2 ^based *meta *p-value. A p-value distribution is generated per replicate following the computation of the one-sided upper tailed Wilcoxon test statistic. A *meta *p-value is generated per site by using a chi-square distribution across all the replicates.

RSSPA is developed upon the framework of replicate statistics. The algorithm has a requirement of a minimum of two replicates per treatment and control and the number of replicates in the treatment and control must be balanced. It does not require a minimum inter-replicate correlation. It is however, important to underscore the impact of the total number versus reproducibility across replicates upon each of the above covariates. The centrality measure in this model is the average (not median) of the ranked pScore; hence it is affected by outliers. The dispersion metric – SAD – ranks sites based on minimization of pair-wise rank differences; for sites with maximal rank consistency, the individual pair-wise rank-differences, and hence SAD will approach 0. Thus independent of the total number of replicates, discordance in ranked pScores will result in the dilution of μ, and inflation in SAD, the consequence being a reduced overall ranking of the putative site. Fig. 1 shows the schematics of the site-level *meta *p-value. *Meta *analysis [31,32] is a multiple comparison approach in which the same (related) hypothesis is tested independently as many times as the available number of replicate experiments, generating a joint p-value (Eqn. 18) and potentially offering more power. Hence for a putative enrichment site even if individual tests are not significant, the joint p-value might still be significant. This test highlights the fact that with increasing number of replicates, the reduced significance obtained from any one comparison has less effect on the outcome of the joint significance. The site-level PVTM (κ = 20%, trimmed mean site-level summary of the Wilcoxon p-value) constitutes the input to the *meta *analysis. The error value term is the reciprocal of the negative log_10 _transformed *meta *p-value. In summary, the performance of the algorithm might be enhanced, if a pre-filtering of replicates is implemented. An all versus all replicate, pair-wise Pearson's correlation coefficient can be computed on the probe-level p-value data and the replicates with lowest correlation coefficient can be eliminated. As discussed, the ChIP on chip assay is prone to artifacts from different noise sources and a minority of probes on the array represents positive enrichment; hence there is inherent noise in the data. In order to demonstrate the effect of noise on RSSPA, results from all replicate experiments irrespective of their degree of discordance have been included.

A cumulative site-likelihood distribution metric (λ) (Eqn. 19) is computed as the resultant of the three p-value based covariates. Specifically, it is computed based on the above three normalized covariates (μ′β,SAD′β,ε′β
 MathType@MTEF@5@5@+=feaafiart1ev1aaatCvAUfKttLearuWrP9MDH5MBPbIqV92AaeXatLxBI9gBaebbnrfifHhDYfgasaacH8akY=wiFfYdH8Gipec8Eeeu0xXdbba9frFj0=OqFfea0dXdd9vqai=hGuQ8kuc9pgc9s8qqaq=dirpe0xb9q8qiLsFr0=vr0=vr0dc8meaabaqaciaacaGaaeqabaqabeGadaaakeaaiiGacuWF8oqBgaqbamaaBaaaleaacqWFYoGyaeqaaOGaeiilaWIaem4uamLaemyqaeKafmiraqKbauaadaWgaaWcbaGae8NSdigabeaakiabcYcaSiqb=v7aLzaafaWaaSbaaSqaaiab=j7aIbqabaaaaa@3AA6@), where each has a [0, 1] bound. Site detection is optimized by simultaneous minimization of the covariates – μ, SAD and ε. Ideal sites are those with high rank, rank consistency, statistical significance, λ approaching 0, and lowest FDR. In most experiments there is clustering of sites based on the distribution of the covariates. A k-means clustering can be employed in this multivariate model space to determine the medoids, inter-cluster and intra-cluster distances. These are useful metrics indicative of the degree of reproducibility across replicates. Rank transformation of each of the above covariates (Eqn. 20) yields overall site-level ranking equivalent to the non-rank transformed case. However, in rank transforming the λ distribution, the inter-cluster distance as well as intra-cluster dispersion data is normalized out and hence lost.

λs=((μ′β,s)2(SAD′β,s)2+(ε′β,s)2)     Eqn.19
 MathType@MTEF@5@5@+=feaafiart1ev1aaatCvAUfKttLearuWrP9MDH5MBPbIqV92AaeXatLxBI9gBaebbnrfifHhDYfgasaacH8akY=wiFfYdH8Gipec8Eeeu0xXdbba9frFj0=OqFfea0dXdd9vqai=hGuQ8kuc9pgc9s8qqaq=dirpe0xb9q8qiLsFr0=vr0=vr0dc8meaabaqaciaacaGaaeqabaqabeGadaaakeaaiiGacqWF7oaBdaWgaaWcbaGaem4Camhabeaakiabg2da9maabmaabaWaaOaaaeaacqGGOaakcuWF8oqBgaqbamaaBaaaleaacqWFYoGycqGGSaalcqWGZbWCaeqaaOGaeiykaKYaaWbaaSqabeaacqaIYaGmaaGcdaqadaqaaiabdofatjabdgeabjqbdseaezaafaWaaSbaaSqaaiab=j7aIjabcYcaSiabdohaZbqabaaakiaawIcacaGLPaaadaahaaWcbeqaaiabikdaYaaakiabgUcaRmaabmaabaGaf8xTduMbauaadaWgaaWcbaGae8NSdiMaeiilaWIaem4CamhabeaaaOGaayjkaiaawMcaamaaCaaaleqabaGaeGOmaidaaaqabaaakiaawIcacaGLPaaacaWLjaGaaCzcaiabbweafjabbghaXjabb6gaUjabc6caUiabigdaXiabiMda5aaa@56E6@

ρλ,s=(Rank(μβ,s)+Rank(SADβ,s)+Rank(εβ,s))     Eqn.20
 MathType@MTEF@5@5@+=feaafiart1ev1aaatCvAUfKttLearuWrP9MDH5MBPbIqV92AaeXatLxBI9gBaebbnrfifHhDYfgasaacH8akY=wiFfYdH8Gipec8Eeeu0xXdbba9frFj0=OqFfea0dXdd9vqai=hGuQ8kuc9pgc9s8qqaq=dirpe0xb9q8qiLsFr0=vr0=vr0dc8meaabaqaciaacaGaaeqabaqabeGadaaakeaaiiGacqWFbpGCdaWgaaWcbaGae83UdWMaeiilaWIaem4Camhabeaakiabg2da9maabmaabaWaaOaaaeaacqWGsbGucqWGHbqycqWGUbGBcqWGRbWAcqGGOaakcqWF8oqBdaWgaaWcbaGae8NSdiMaeiilaWIaem4CamhabeaakiabcMcaPiabgUcaRiabdkfasjabdggaHjabd6gaUjabdUgaRjabcIcaOiabdofatjabdgeabjabdseaenaaBaaaleaacqWFYoGycqGGSaalcqWGZbWCaeqaaOGaeiykaKIaey4kaSIaemOuaiLaemyyaeMaemOBa4Maem4AaSMaeiikaGIae8xTdu2aaSbaaSqaaiab=j7aIjabcYcaSiabdohaZbqabaGccqGGPaqkaSqabaaakiaawIcacaGLPaaacaWLjaGaaCzcaiabbweafjabbghaXjabb6gaUjabc6caUiabikdaYiabicdaWaaa@66CF@

The λ distribution can be used for final ranking and segmentation of the sites. If reproducibility is of primary concern, maximally consistent and reproducible sites belonging to the lowest percentiles of the λ distribution can be selected for further investigation. If all sites are to be considered for further investigation, the λ-based distribution enables binning of sites based on detection enrichment and confidence. In most cases, however, before multivariate analysis can be performed the missing data problem discussed below must be addressed.

#### The missing data problem

In an ideal model, following initial segmentation, identical site intervals should be detected across all replicates (Eqn. 21). The rationale behind the ideal model is that in all biological replicate samples, any given enrichment site should be identified by the exact same sequence; hence the probe to site relationship should remain constant. In reality there exists discordance in the distribution of site intervals (Eqn. 22); this is expected in samples derived from different biological replicates or cell growths, which might not be in synchronized states, and/or in technical replicates, due to hybridization variations. Cumulative errors from sources of variation in the experimental pipeline result in variable degrees of immunoprecipitation, which is the root cause of the site interval distribution not conforming to the idealized model. In summary, the frequency of the seed sites might not be identical and/or the site intervals might not be equivalent across replicates. While errors arising from partial overlap of sites could be mitigated by estimating the peak position of the enrichment activity, the complete absence of sites from some replicates causes a missing data problem. This is addressed by assigning the sites absent – in any replicate – with a surrogate or missing data value (MDV). The MDV is a constant for a set of replicate datasets and it corresponds to the rank exceeding the maximum rank across all replicates as shown in Eqn. 23. The MDV down-weights the surrogate site in the computational process.

**S **= *S*_1 _= *S*_2 _= ... = *S*_*r *_= *S*_1 _∩ *S*_2 _... ∩ *S*_*r *_*where replicates*: 1 ≥ *r *≥ *R and S: collection of sites in any given r *    Eqn. 21

**i) **(*S*_1 _∩ *S*_2 _... ∩ *S*_*r*_) ⊂ **S **and **ii) **(*S*_1 _∪ *S*_2 _... ∪ *S*_*r*_) ≥ **S**.     Eqn. 22

*MDV *= 1 + max(max(*ρ*_1_),max(*ρ*_2_), ..., max(*ρ*_*r*_))     Eqn. 23

#### Step 3: Final segmentation of sites based on a stringent signal enrichment threshold

The final parameter in this model, SE measures the relative enrichment in the treated sample with respect to the control. The reported signal enrichment is a robust estimate – site-level median as aggregated across all replicates and is defined as: *median*(*SETM*)_*s*_. In the event of seeding sites based on a p-value threshold (τ_p_) there is no expected minimum *median*(*SETM*) for any site. However, in the event of seeding based on SE threshold (τ_*s*_), the *median*(*SETM*) for a site might be less than τ_*s *_– the expected minimum. This is primarily due to two reasons. First, the seeding process occurs independently in each replicate. But the ultimate ranking and prediction is based on a consensus measure of the presence of any given site in the majority of replicates. This implies that in a subset of replicates (r < R), the measured SE for a given site could approach 0, resulting in the *median*(*SETM*) less than τ_*s*_. Second, due to fragmentation in the site interval introduced by the localization of site boundaries (discussed below), the final site might encompass only a subset of probes, in contrast to the original probe membership for that site for a given replicate. To guard against over-fragmentation and also against false positives, sites belonging to the λ distribution are filtered by *median*(*SETM*) ≥ *τ*_*s*_/*R*. This operation results in a final set of sites: *λ*_*s *_⊆ *λ*;

The final outcome of the algorithm is either a ranked list of predicted sites based on the ranked λ_s _distribution, or a thresholded list of predicted sites based on the *meta *p-value and/or *median*(*SETM*). An alterative method of segmentation based on FDR which serves the dual purpose of providing correction for multiple hypothesis testing, has been applied. The FDRs are generated empirically from the data based on the method published by Efron [33]. In contrast to the more conservative Bonferroni correction [34] FDR is more appropriate for analysis of ChIP on chip data where non-canonical sites might exhibit reduced levels of enrichment and significance in comparison to their canonical counterparts. FDR-based segmentation is particularly useful for comparison of data generated across different ChIP on chip platforms, where the stringency of the FDR, as tuned to each individual platform, is maintained constant across all platforms.

#### Step 4: Localization of site boundaries

There are two contrasting approaches for generation of final site-interval in genomic space. i) Greedy: For a given site the *union *of the site-intervals across all replicates is considered. (Eqn. 24).  ii) Conservative: For a given site the *intersection *of the site-intervals across all replicates is considered (Eqn. 25). Physically, this results in the localization of the enrichment peak rather than in exact delineation of the change-points. *Sitelnterval*_*s *_= (*B*_1 _(*start*, *stop*)∪ *B*_2_(*start*, *stop*)... ∪ ... *B*_*r *_(*start*, *stop*))_*s *_    Eqn. 24 *Sitelnterval*_*s *_= (*B*_*1*_(*start*, *stop*)∩ *B*_2_(*start*, *stop*)... ∩ ... *B*_*r *_(*start*, *stop*))_*s *_    Eqn. 25 At the seeding stage, the probe membership for a given site can vary across replicates. At the site localization stage, probes with a dominant pattern of co-regulation are clustered together to generate the final site interval. The SETM is evaluated subsequent to site localization. For sites with diminishing reproducibility the final probe cluster might be significantly reduced compared to the union of the initial probe clusters; this could result in a SETM much less than τ_*s*_.

## Results

Results of the application of RSSPA for detection of histone acetylation and RNA polymerase II occupancy sites are described here. The experiments performed in the HL-60 – an acute myeloid leukemia – cell-line, explore the interaction of DNA with (i) tetra-acetylated histone (HisH4), and (ii) RNA pol II [35]. HL60 is stimulated with all-trans-retinoic acid for distinct time periods, to induce differentiation along the granulocytic lineage. 0, 2, 8 and 32 hrs constitute the time-course in this experimental design. The site prediction algorithm is applied per time-point and differential modification analyses (data not presented here) are performed subsequently. HisH4 is a histone modification factor associated with active genes. RNA pol II is the nuclear RNA polymerase responsible for mRNA transcription. In eukaryotes, unlike prokaryotes, the normal or ground state of the chromatin is restrictive to transcription. In the repressed state the enhancer and promoter elements are covered by nucleosomes. This state can be converted, via acetylation, methylation and recruitment of chromatin remodeling factors, into a transcriptionally poised state that is prepared for binding to RNA pol II and TFIID proteins [36]. Both HisH4 and RNA pol II are hybridized to Affymetrix ENCODE [26,27] tiling arrays of 22 bp (average) probe resolution and 10μ feature resolution. The ENCODE array samples approximately 1% of the human genome and does not include regions from chromosomes 3 and 17.

RSSPA has been implemented for detection, ranking and segmentation of enrichment sites across a spectrum of ChIP on chip experiments. These range from chromatin remodeling factor (Brg1), sequence-specific DNA binding proteins (CTCF, CEBP/ε), histone modification factors (HisH4: acetylation, H3K9K14D, H3K27T: methylation), to factors with known 5' end biases (RNA pol II, TFIID). Data on the above factors are being published as part of the ENCODE Consortium effort [37,38]. The HisH4 and RNA pol II data, a subset of the ENCODE data, are discussed here since they represent contrasting enrichment profiles – in terms of the base pair coverage of their binding footprints. The results will focus on:

i) Common parametric approaches for detection of enrichment sites

ii) The underlying mechanism and efficacy of the proposed non-parametric RSSPA

iii) Simulation results

iv) Biological examples

v) Validation results derived from alternative computational and biochemical approaches

### Binary segmentation for detection of enrichment sites

This simple, intuitive approach validated via quantitative PCR (qPCR), is effective in the identification of sites with relatively strong enrichment signal and statistical confidence. While ensuring low false positive rates, this method can suffer from a significant false negative bias, especially in regions of diminished signal enrichment. A span of DNA sequence is computationally labeled a non-site (negative) if it fails the stipulated signal or p-value threshold. This binary outcome does not reveal whether the absence of a site is due to a potential false negative caused by failing the threshold by a minor margin, or is a true negative caused by a span of DNA with very low significance and negligible IP enrichment.

**Figure 2:**
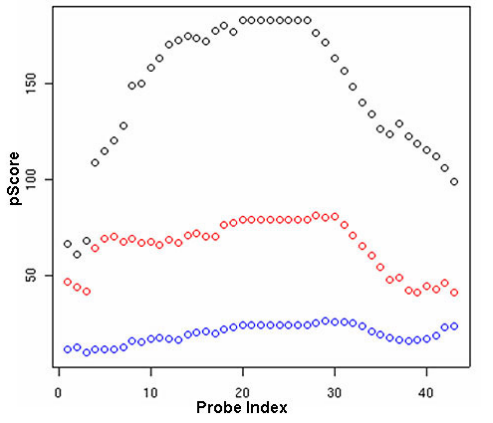
Biases in the parametric binary segmentation method for detection of putative enrichment sites-I. pScore distribution across 43 consecutive probes in three different biological (B_1_-B_3_) replicates, are shown. Probes belonging to B_1 _(black) and B_2 _(red) pass the pScore threshold of 50 while those belonging to B_3 _(blue) fail by a significant margin. The trend in the pScore distribution hints at potential enrichment but if B_3 _were the only dataset available a false negative bias could have been introduced in the analysis.

The intrinsic noise in ChIP on chip can reduce the SNR in the data and result in a globally lower p-value and/or signal enrichment distributions. In these circumstances, a computationally determined negative might be positively validated by qPCR and/or other biochemical means. Thus in experiments with high variance and/or reduced binding efficiency, a significant false negative bias can be introduced, resulting in inflated discordance across replicate experiments, as demonstrated by Fig. 2. The figure shows, for three replicates, the pScore profile across 43 contiguous positive probes that constitute a putative site. At this site only two thirds of the biological replicates exceed the pScore threshold of 50(Wilcoxon p-value = 10^-5^) for a set of contiguous probes, potentially indicating variable levels of sensitivity in the experiments. The overall pScore trend is consistent across replicates, hinting at the presence of a putative site. However, if the replicate with the lowest pScore distribution (blue curve) were the only dataset available and the binary segmentation with a pScore threshold of 40, the method of choice then this site would become a false negative. In the absence of independent corroboration it would be difficult to discriminate the putative target (in the lowest sensitivity experiment) from an artifact of spatial auto-correlation.

**Figure 3:**
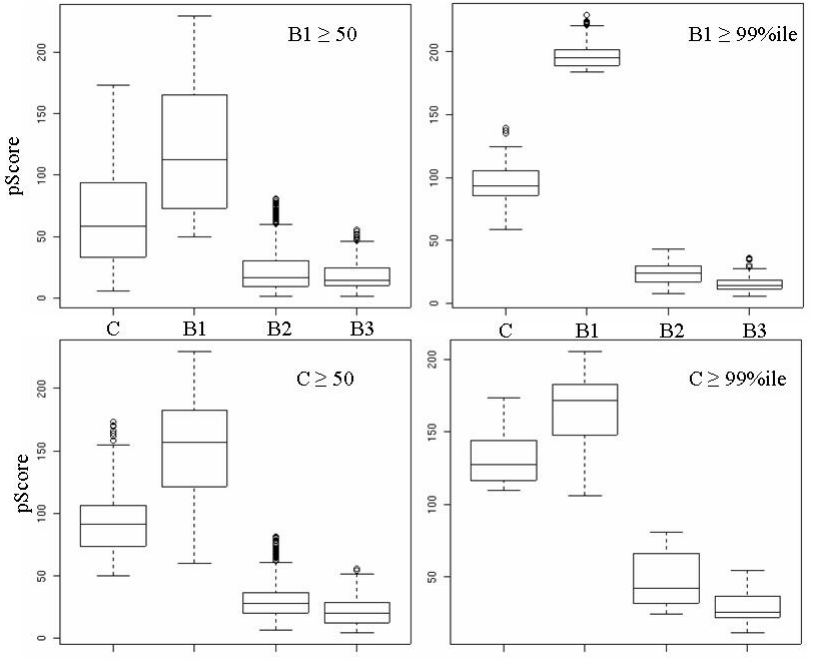
Biases in the parametric binary segmentation method for detection of putative enrichment sites-II. This demonstrates a comparison of site detection in B_1_-_3 _and replicate composite(C) based on the threshold selection. Depending on the threshold and which replicate it is determined from, there is significant variation in the detection of putative sites. (Top panel) Detection threshold is determined based on B_1 _but applied uniformly across B_1_-B_3 _and replicate composite (C); (left) Fixed pScore threshold of σ_p _≥ 50; (right) A distributional threshold of σ_p _≥ 99^th ^percentile of the pScore distribution. (Bottom panel) Detection threshold is determined based on replicate composite(C) but applied uniformly to all; (left) Fixed pScore threshold of σ_p _≥ 50; (right) A distributional threshold of σ_p _≥ 99^th> ^percentile of the pScore distribution.

Threshold estimation controls the sensitivity versus specificity of the binary segmentation approach. Each of the thresholding mechanisms – whether of fixed or distributional type – introduces a different type of bias to the analysis, examples of which are shown in Fig. 3. The figure shows box-plots of the pScore distribution of three biological replicates (B_1_-B_3_) and their replicate composite, following binary segmentation. Fig. 3 (upper panel) contrasts site detection based on a fixed versus distributional pScore threshold derived from replicate B_1_. The two pScore thresholds used are: (a) fixed: 50 (pScore ≥ 50); and (b) distributional: 99^th ^percentile of the B_1 _distribution. Once the distributional threshold is derived from a particular replicate, the same value is applied uniformly across all replicates. The binary segmentation after fixed thresholding show that the sites in B_1 _have a minimum score of 50 (as expected), while the pScore distribution of these exact sites in replicates B_2_, B_3_, and the composite, manifest a significant range from 0–200. For the distribution-derived threshold, the detected sites have a pScore range of 170–230(B_1_), 0–50 (B_2_-B_3_), and 50–150 (composite). This result highlights disparity in the score-distribution across replicate biological samples and demonstrates that fixed and distribution-derived thresholds might not detect identical sites across replicates, resulting in increased disparity among replicate experiments. An alternative to using individual replicates is to generate the fixed/distributional thresholds based on the replicate composite. But, as shown in Fig 3 (lower panel), this hardly mitigates the disparity. While the choice of a composite over an individual replicate does not improve the performance of the method, the choice of a distributional over a fixed threshold reduces the variance in the pScore distribution of the putative sites. This is evident from the maximal compression of the inter-quartile range observed in Fig 3 (top-right panel).

### Rank statistics based algorithm for detection of enrichment sites

A parametric binary segmentation paradigm has the potential to introduce a significant false negative bias. This bias discriminates against sites with moderate to low binding-enrichment, or poor probe behavior. RSSPA employs a rank and replicate statistics-based paradigm to mitigate these biases. The following sections discuss the results from each of the components of RSSPA.

#### Step 1 – Seeding of sites based on binary segmentation of data

**Figure 4:**
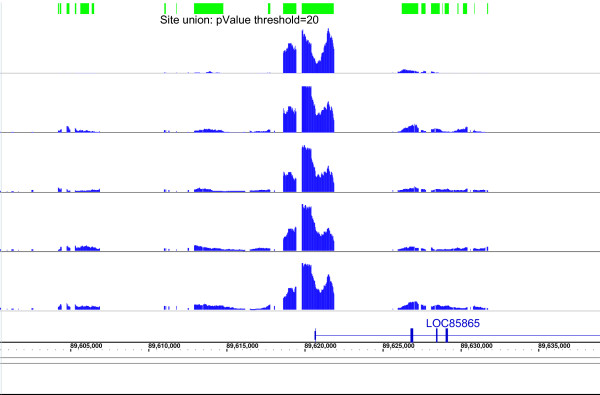
*Seeding of sites *using a pScore based threshold. Representative data is shown for the *site-seeding *step based on a pScore threshold of 20. A threshold of 20 results in an approximate minimum SNR of 1.1. Sites are generated individually in each of the replicate datasets. In this example, the pScore graphs in blue represent five replicates of HisH4. The top-most track in green represents the union of the site-intervals as derived from each of the replicates.

Results have been generated based on both seeding parameters – p-value and SE. The site seeding is potentially more robust if based on signal enrichment, rather than p-value distribution. Since the latter is affected more significantly by spatial auto-correlation. The final results summarize the correlation obtained between the two seeding parameters. Fig. 4 shows an example of site-seeding, based on a pScore threshold of 20, across five biological replicates in the HisH4 data. The amplitude of the graphs (blue) represents the pScore distribution in a specific genomic region where all replicates manifest similar ChIP-enrichment (all graphs have been scaled to common maximum for clarity of visual representation). The top-most track (green) represents the union of the site intervals as derived from the individual replicates.

#### Step 2 – Optimization of sites based on centrality, variance and error distributions

**Figure 5:**
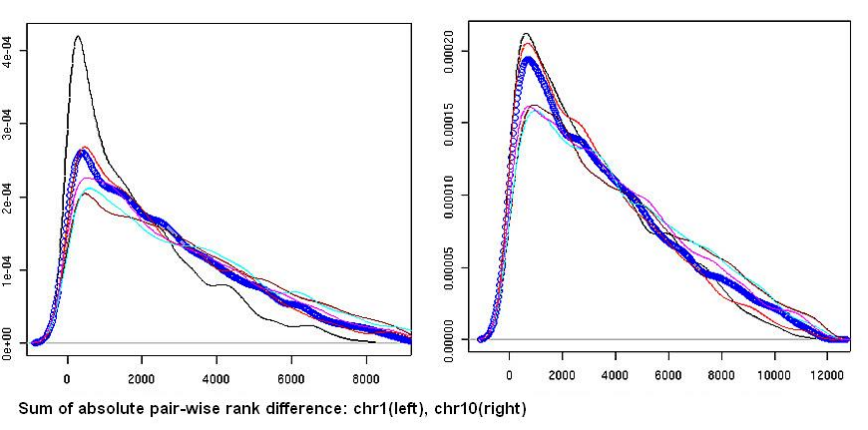
Pair-wise distribution of absolute rank-differences across replicate datasets. The absolute rank-difference comparisons are shown for all pair-wise permutations of replicates (B_1_-B_3_) and replicate composite (C). C-B2(blue);C-B3(red);Bl-B2(brown);Bl-B3(cyan);B2-B3(magenta) represent the different pairs. Distributions are shown for two different chromosomal segments with contrasting gene density: (left) Chromosome 1: poor gene density (right) Chromosome 10: high gene density.

The optimization based on the simultaneous minimization of the p-value based covariates – μ, SAD and ε – and maximization of SE forms the corner-stone of the algorithm (Steps 2–3). Fig. 5 summarizes the rank consistency distribution for putative sites. It demonstrates density plots of pair-wise rank-difference distributions for replicate data derived from chromosomal segments with contrasting gene density. Poor gene density (chromosome 1) is shown at left, higher gene density (chromosome 10) is shown at right. Based on triplicate experiments, the absolute rank-difference distribution is computed for all six pair-wise replicates – {B_i_, B_j_} and {C,B_i_} – where, i and j refer to replicates, l ≥ i(j) ≥ 3 and i ≠ j, and the replicate composite (C). Data for the C-B_1 _pair is shown in black, C-B_2 _in blue, C-B_3 _in red, B_1_-B_2 _in brown, B_1_-B_3 _in cyan and B_2_-B_3 _in magenta. For visual clarity, the x-axis is scaled by 100, data-points are shown for one curve (C-B_2_) and for others a spline-fit to the data has been represented. The primary observations here are:

i) The absolute rank-difference curves do not trace a delta function about 0 (ideal case) or even an exponential decay with a peak at 0, but rather a gamma distribution with the mode slightly greater than 0. This observation holds true across all chromosomal regions. The off-zero mode indicates that very few sites show perfect rank consistency. This is due to inherent noise in the ChIP on chip process. However, the bulk of the population of seed sites maintains very high rank consistency across replicates. This validates a fundamental assumption of the model.

ii) The skewness of the distribution is attributable to the population of sites whose rank order correlation across replicates gradually diminishes. More than 95 percent of these sites have a high likelihood of inherently poor intra-replicate ranking (data not shown).

iii) The SAD distribution is estimated based on seeded sites which include all possible ChIP enrichment intervals that exceed a SNR of 1.1. Potentially a high percentage of these sites could be false discoveries. The FDR could be reduced by re-adjusting the parameters of the normal distribution (N(μ,σ^2^)) based on the underlying gamma distribution:

(a) Setting the estimated mean (μ) to the mode of the gamma distribution;

(b) Estimating the variance (σ) by symmetrizing the left tail of the gamma distribution.

The proposed modification in the estimation of the normal distribution would filter out sites with high SAD values. In order to explore the response of RSSPA to various noise sources, results presented here do not incorporate this correction.

iv) Independent of gene density – as observed from data across both panels – there is a strong correlation (R^2 ^≥ 0.87) across all pair-wise absolute rank difference distributions considered. The contrasting gene density data demonstrates that the correlation in the rank-difference profiles is maximal in the gene poor regions (R^2 ^≥ 0.94). The reduced correlation in the gene rich regions is potentially due to the fact that the variable sensitivity in ChIP on chip experiments has maximal impact here. Overall, the rank order preservation in sites is strongest for the pair-wise combination of C-B_1_. This observation reflects the fact that the pseudo-median replicate composite distribution is dominated by the B_1 _replicate profile, which is the experiment with highest sensitivity.

#### Step 3: Final segmentation of sites based on a stringent signal enrichment threshold

**Figure 6:**
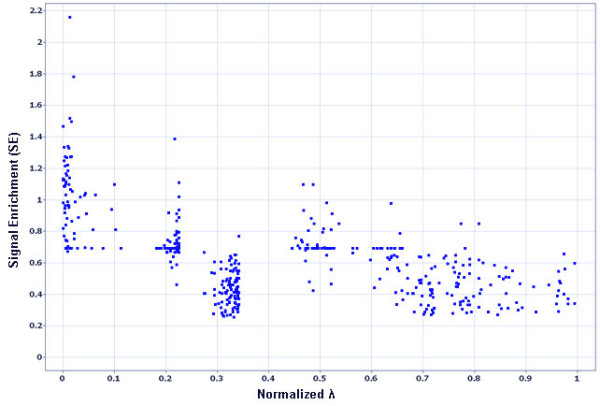
Distribution of λ versus signal enrichment (SE) for segmented sites. Sites with minimal λ (x-axis) show enhanced signal enrichment or SE (y-axis). Sites with maximal signal enrichment are generally contained in the top 25^th ^percentile of the λ distribution.

RSSPA optimizes site-detection based on simultaneous minimization of λ and maximization of SE, demonstrated in Fig. 6. Sites in the λ distribution, that manifest at least two-fold immunoprecipitated enrichment in at least one of the replicates, are considered candidate sites, λ_s_, for the final ranking and segmentation. The two-fold IP enrichment threshold is assessed based on the lowest detection limits of qPCR. Sites with the highest enrichment generally populate the first quartile of the λ distribution (Fig. 6). qPCR validation results discussed below show that a two-fold array enrichment threshold is indeed stringent, since the dynamic range of a microarray measurement is compressed in comparison to that of qPCR measurements. The stringency of the SE threshold is user tunable and can be altogether omitted, depending upon the specificity required. The algorithm performance subsequent to the application of the above-mentioned filters has been discussed under *method validation*.

### Simulation results

**Figure 7:**
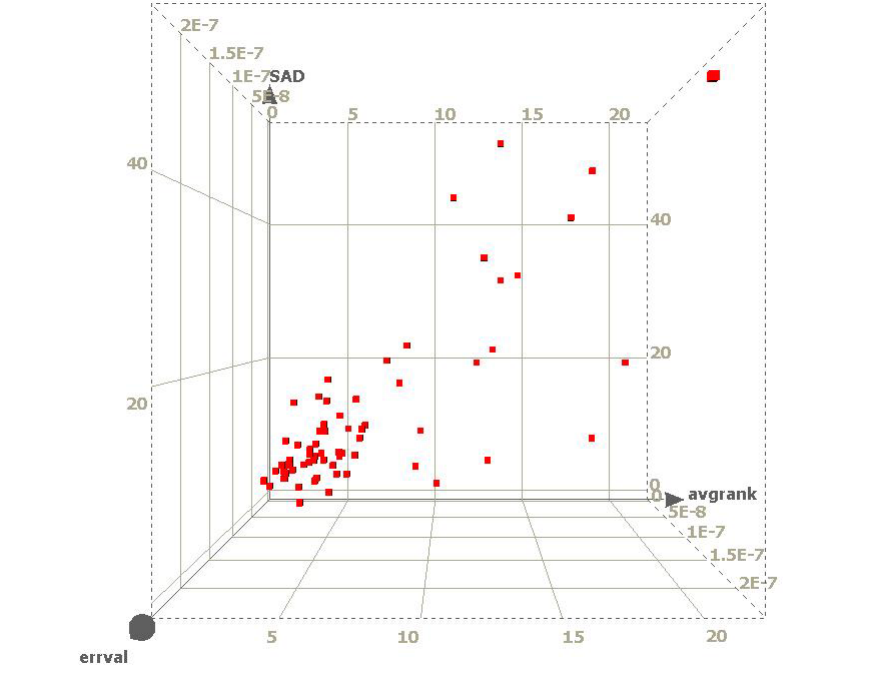
Simulation Results. A simulation demonstrating the distribution of the three p-value derived parameters: μ or average rank (x-axis), SAD (y-axis) and ε or errval (z-axis) for 100 sites. The sites are generated based on four replicate datasets with significant reproducibility (Spearman's ρ = 0.93). The seeding is based on the p-value distributions. The ideal sites with maximal rank consistency are represented by the vertex cluster at [μ, SAD, ε] = [0,0,0]. Scatter is introduced in the dataset via artificial introduction of noise. This causes gradual migration of sites from the vertex cluster to the top right hand edge of the graph, where the sites represent the worst intra-replicate ranks, least inter-replicate rank consistency and lowest statistical significance. Despite the introduction of significant level of noise, the density of the vertex cluster remains the highest.

The simulation results show a progression of RSSPA response obtained from data with very high SNR to data with artificially introduced noise (lower SNR). Fig. 7 shows a simulation result for 100 sites, derived from quadruplicate datasets with significant reproducibility – Spearman's ρ of approximately 0.93. In this simulation the sites are seeded based on the p-value distribution. The three axes of the figure represent the covariates: μ, (x-axis), ε or errval (y-axis) and SAD (z-axis). Following the minimization optimization, a high-density cluster is observed around the vertex (μ = 0, SAD = 0, ε = 0). The vertex cluster represents the sites with maximal intra-replicate ranks and inter-replicate rank consistency and highest statistical confidence. Since the dataset is simulated for a high SNR condition, the diminishing cluster density away from the minima is expected.

In order to test the efficacy of the algorithm, variable levels of outliers are simulated by the introduction of correlated noise in the data. The results show that monitoring of inter-cluster and intra-cluster metrics allow users to dynamically assess reproducibility across replicate experiments. With decreasing SNR the cluster density migrates from the minima (vertex) to the top right where the errors on covariates are maximized. Experiments with highest reproducibility result in a vertex cluster with maximum density and minimum intra-cluster variance. Based on analysis of several ChIP factors, it has been determined that for experiments with greater than one cluster, vertex clusters with a density of 90 percent or higher (90 percent or more of all sites detected occupy the vertex cluster) and maximum vertex cluster radius of less than 1.5 times vertex-cluster standard deviation, manifest a Spearman's ρ of greater than 0.92 and generally have a FDR of less than or equal to 5 percent. By maintaining the number of replicates constant and varying reproducibility, there occurs a migration of sites away from the vertex cluster and an increase in the intra-cluster variance. This highlights the consequence of reduced reproducibility in a ChIP on chip experiment. This class of vertex-cluster sites can provide anchor points for experimentalists to perform further biological validation including qPCR to investigate the dynamics of transcriptional regulation. A study of change in the membership of the vertex cluster, in a time course experiment, is a powerful tool to probe the differential changes in cells subject to external stimuli.

### Biological examples

**Figure 8:**
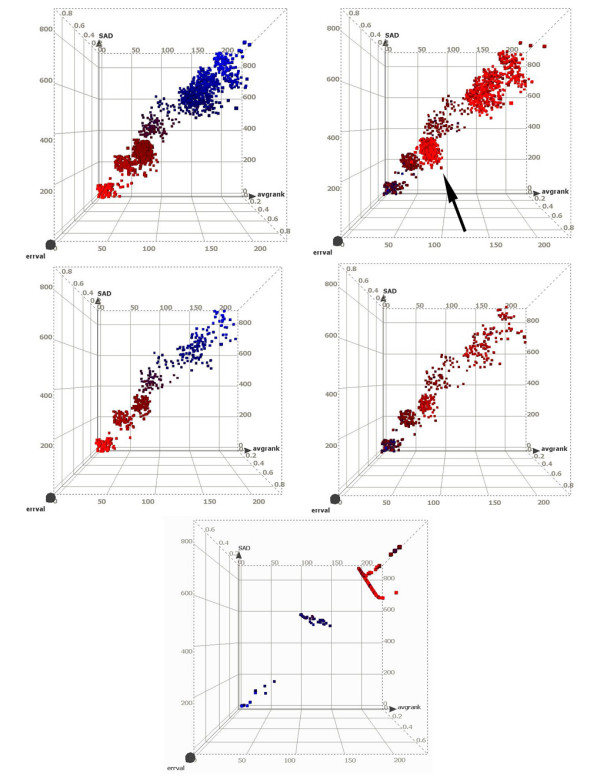
Rank Statistics based site prediction algorithm outcome for RNA pol II. Distribution of RNA pol II sites in 3 parameter p-value space (μ, SAD, ε) with the color-map indicating ranking of sites based on λ, distribution (left); SE distribution (right). This distribution is computed for data from 5 replicates. Color-map: blue and red represents the maximum and minimum bounds of the respective distributions. (Top panels) The p-value centric 3 component ranking has concordance with the SE based ranking, except for the cluster indicated by an arrow. (Center panels) Removal of sites belonging to the aberrant cluster increases the rank correlation from 81.2% to 97.5%. These sites are eliminated following the application of a SE based filter, where the SE threshold is based on the median (SETM) of the aberrant cluster. (Bottom panels) Unlike the prior distribution, this dataset reflect the presence of three distinct site clusters. This output is based on the maximally discordant triplicate experiments of the above five replicates.

The first set of results is presented for the occupancy of RNA pol II as determined in the HL60 cell line. The experimental design comprises five biological replicates, each with a single technical replicate (5 × 1), yielding a total of ten datasets across IP (five replicates) and control (five replicates). Wilcoxon-p-value and a HL-based signal estimate distributions using a sliding window of 1 kb are computed per replicate. The results of RSSPA for five replicate pairs are shown in Fig. 8 (top and center panels). Each plot shows three axes representative of μ (x-axis), ε (y-axis) and SAD (z-axis). The color map represents the gradient of the site distribution based on λ (left panels) and *SE *(right panels), with the maximum and minimum denoted by blue and red respectively. Sites with lowest FDR occupy the minimum end of the λ spectrum (left panels) and the maximum end of the SE (right panels) spectrum. The primary observations here are:

i) Site distribution is along a continuum, rather than in isolated clusters.

ii) The ranking distributions in λ and SE show an overall strong correlation of Spearman's ρ of approximately 0.812. However, there is also a distinctly aberrant cluster (indicated by the arrow in the top right panel).

The following are two potential explanations of the above observations. First – while the replicates are not perfect – as evident from outliers in the λ distribution – their overall distributions are relatively similar. If the inter-replicate distributions were significantly different they would separate into discrete clusters highlighting the concordance across some and discordance across others. Second – in order to understand the origin of the aberrant cluster, the continuum is segmented into percentiles based on *SE*. A Euclidean distance metric is computed across the medoids of the percentile bins; this localizes the aberrant cluster. The aberrant cluster has maximal similarity to the cluster with lowest SE but its p-value-based metrics ascribe it a higher statistical confidence. This may be an artifact of auto-correlation contamination of the p-value. This cluster is eliminated (bottom panels) by applying the stringent overall SE filter of *median*(*SETM*_*s*_) > 0.693/*R *(R: maximum number of replicates). Following this elimination, the Spearman's ρ correlation between SE and λ improves from 0.812 to 0.975. The power of this approach is that it enables extraction of sub-optimal sites, albeit with a lower consistency score, whose presence might not be reproducible across replicates.

The following analysis highlights the impact of reproducibility across replicate experiments on the assessment of ChIP-enriched sites. In Fig. 8 (bottom panel), data for three out of five replicates in RNA pol II is summarized. The replicates are chosen on the basis of least pair-wise reproducibility. The plot shows the components of the λ distribution along the three axes: μ (x-axis), ε (y-axis) and SAD (z-axis). The color-map shows the ranking based on SE with blue and red corresponding to the maximum and minimum respectively. The primary observations here are:

i) Unlike the prior result, where the site distribution followed a continuum, here there are three distinct clusters generated primarily in response to the discordance across replicates.

(a) Cluster I is the vertex cluster with μ, SAD and ε approaching 0.

(b) Cluster II represents sites with ranks in the inter-quartile range of each replicate and whose rank order may be discordant in a subset (1/3 or 2/3) of replicates.

(c) Cluster III, the most distal one, reflects sites with ranks in the uppermost quartile within each replicate. These sites are those with maximum discordance in rank order across replicates. The discordance in the rank ordering is potentially introduced by variability in sensitivity, for example, the case where a replicate IP array is significantly more sensitive than the other two. This variability in sensitivity has maximal impact on chromatin modification sites that have an overall lower level of expression of modification.

ii) Increasing intra-cluster dispersion is observed in the more distal clusters.

iii) The segmentation based on ranked p-value metrics has > 99 percent concordance with the segmentation based on ranked signal enrichment, as shown via the color-map. This indicates that there is internal agreement for the ordering of sites based on the three p-value-centric covariates as well as with the ordering based on SE.

**Figure 9:**
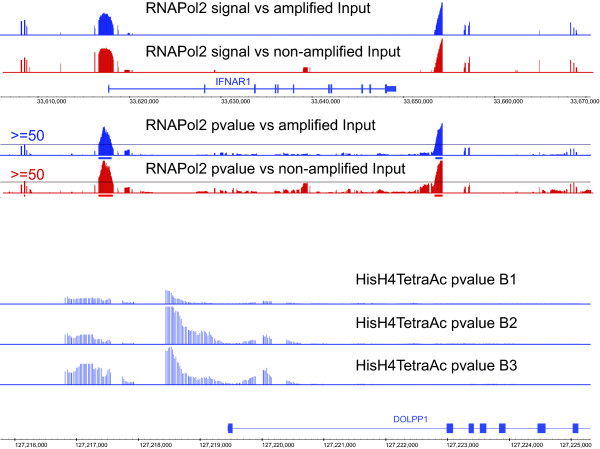
Comparison of the enrichment profiles for RNA pol II (top panel) and HisH4 (top panel). Data represents the signal (upper two tracks) and p-value (lower two tracks) enrichment for RNA pol II and p-value enrichment for HisH4.

Fig. 9 (top panel) shows an example region contrasting the array signal (top two tracks) and p-value enrichment (bottom two tracks) profiles for RNA pol II. Data from two biological replicates (generated with respect to amplified and non-amplified inputs) at the 00 hr are shown. The x-axis represents the genomic coordinate and the y-axes represent the amplitude of SE and pScore, with tracks scaled to their respective distribution bounds to facilitate visual data comparison. In this panel the distance between tick marks on the x-axis is l0 kbp. The observations from the data are the following:

i) Visually, there exists a strong concordance between the pScore and SE distributions. For the replicates the concordance ranges between 0.97–0.99(data not shown).

ii) There is a strong concordance between the putative sites independent of whether the seeding is based on pScore or SE. Based on analysis across all five replicates an 89 percent bp intersection is observed between putative sites seeded by pScore or SE.

iii) Two distinct sites (blue and red) are observed here. One of the sites overlap with the 5' end of the IFNAR1 gene and the other is approximately 3000 bp downstream of the 3'end. The span of the sites range from 800–1100 bp.

HisH4 with a broader distribution across the interrogated parts of the genome presents a significantly different enrichment footprint in comparison to RNA pol II. The acetylation regions often span 1 kb-long (or longer) genomic sequences, and are frequently observed as enrichment plateaus rather than as peaks. Fig. 9 (bottom panel) shows an example region contrasting the p-value enrichment profile of HisH4 in three biological replicates (B_1_-B_3_) at the 00 hr. The x-axis is representative of the genomic coordinate and the y-axis or amplitude of the graphs is representative of pScore with tracks scaled to the same bounds to facilitate visual data comparison. Also in this panel the distance between tick marks on the x-axis is 1 kbp. All three replicates show evidence of a pair of ChIP-enriched sites, one slightly upstream and the other overlapping with the 5'end of the DOLPP1 gene on chromosome 9. The putative site upstream of the annotation has a footprint of 750–850 bp, whereas the one overlapping with the 5'end has a footprint of 1800–2000 bp. It is conceivable that the fragmentation in the site is due to the presence of interspersed repeat sequences that have not been tiled; hence in truth it is a single site spanning over 3 kb. Nonetheless, the positive probes contributing to the creation of the site exhibit a highly concordant ChIP enrichment profile over a significant span of the sequence. This represents a footprint very different from sequence specific factors where motif identification might be stronger predictors of target sites. HisH4 exhibits a basal level of acetylation, with plateaus rising above the baseline, representing longer periods of persistence in an acetylated state. This is in contrast is to RNA pol II potentially because the binding occupancy of RNA pol II emulates a switch with two discrete states – bound and unbound. The efficacy of RSSPA for detecting enrichment profiles, irrespective of a site's span, is discussed in the *method validation*. RSSPA's independence to the span of binding activity is mainly because the model is not shape-based; instead, it utilizes the concordant behavior within a neighborhood of contiguous probes, and consistency of probe behavior across replicate experiments.

Site predictions for both factors were segmented at 1, 5 and 10 percent FDR. The median overlap of predicted sites of RNA pol II occupancy and HisH4 acetylation at 1 and 10 percent FDR was 42.7 and 50.89 percent respectively. The median is generated from the time-course experimental dataset (time-points of 0, 2, 8 and 32 hours). The standard deviation in the overlap across the time-course is 7 percent. These observations indicate that, despite the differences in the enrichment profiles, there is significant recapitulation of sites across both factors – this in itself is a validation of the performance of RSPPA. Approximately 84 percent of the site overlap that occurs is significant at 1 percent FDR. The qPCR validation results (below) show significant concordance with site prediction at the level of 5 percent FDR.

### Method validation

**Figure 10:**
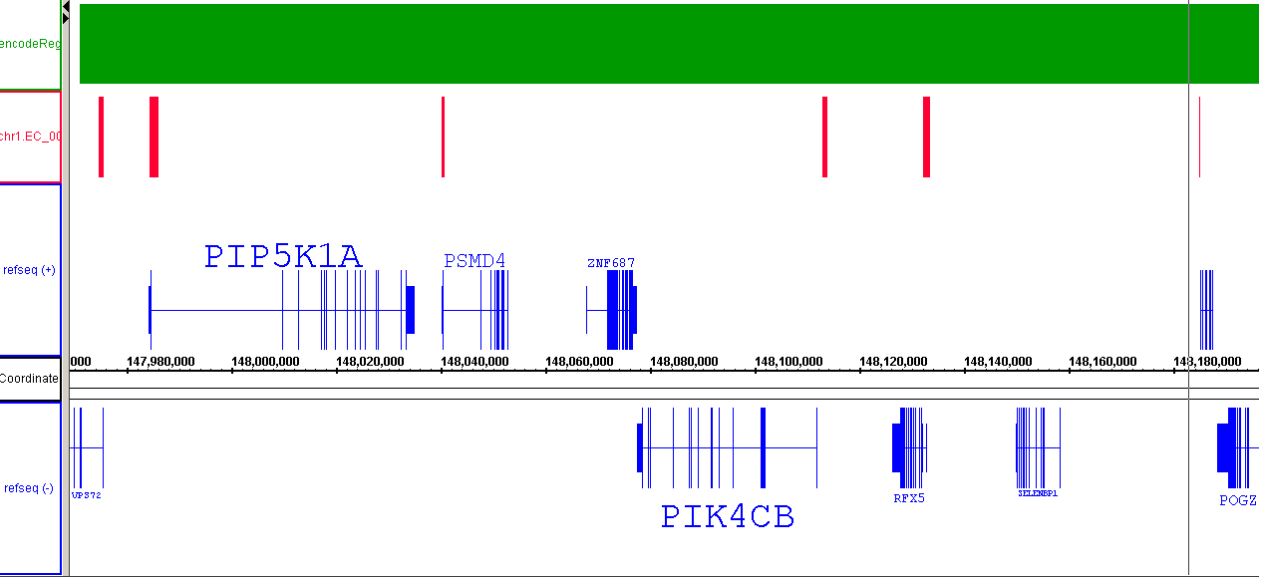
Validation of RSSPA using annotation data. Overlap of a set of RNA pol II sites with 5'ends of known annotation on both the sense and anti-sense strands. Representative data is shown for chromosome 1 where the overlap occurs with both PIP5K1A and PSMD4 and TCFL1 (VPS72) on the sense and anti-sense strands respectively. The 5'enrichment of RNA pol II is significant at p < 0.0001 as established by bootstrapping.

Two types of validation data are presented. In the first type, the performance characterization of RSSPA is based upon statistical techniques and in the second type it is based upon validation with respect to qPCR. In the statistical approaches, knowledge of RNA pol II enrichment for the 5' ends of transcripts has been utilized. The significance of the overlap of RNA pol II sites with the 5'ends of known annotation (RefSeq and VEGA) is estimated via boot-strapping. The significance of the 5' enrichment is computed to be p < 0.000l. Fig. 10 shows the overlap of a subset of RNA pol II sites, of span 500–1400 bp, with the 5'ends of known annotation. The x-axis refers to genomic coordinates with the four tracks representing annotation. The track in red is representative of the predicted site intervals; the tracks in blue are representative of RefSeq annotations along the sense and anti-sense strands indicated by RefSeq(+) and RefSeq(-) respectively. The RefSeq(+) track is aligned along the 5' to 3' direction with the RefSeq(-) track being vice-versa; the track in green is representative of the coverage of the ENCODE region on the array. This particular visualization shows RNA pol II sites overlapping the 5'end of transcripts – PIP5K1A, PSMD4 – on the sense strand and TCFL(alias: VPS72), PIK4CB on the anti-sense strand. Sites are also found internal to transcripts, and in intergenic space (data not shown). The presence of these sites can be validated with qPCR, but ascertaining their biological significance – whether they hint at poised or paused states of RNA pol II transcription machinery – requires further biological experimentation.

**Figure 11:**
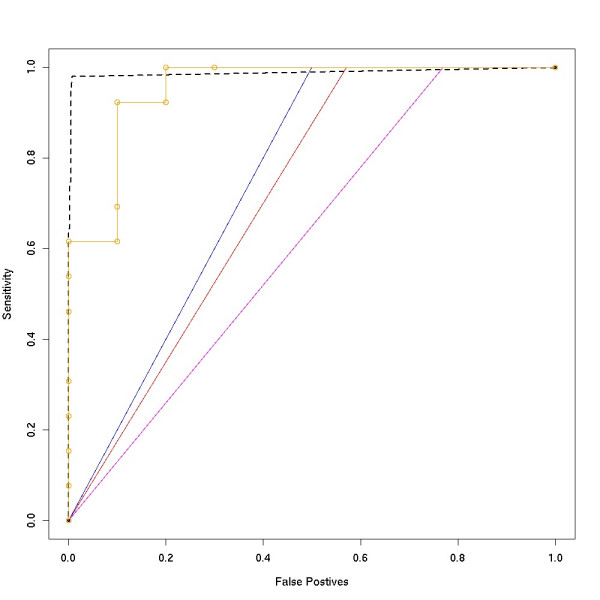
Performance of RSSPA. A pseudo ROC method is employed here for assessment of the performance of RSSPA versus binary segmentation. The x-axis corresponds to the FPR(1 – specificity) and the y-axis corresponds to sensitivity. The solid curves are representative of RNA pol II data derived from chromosome 1 (ENCODE array) and the dotted curve is representative of RNA pol II data derived from all chromosomes sampled by the ENCODE array. The solid curves exhibit the performance of RSSPA (orange) versus binary segmentation using a pScore of 50 (blue), 40 (red) and 30 (magenta). The dotted curve represents the performance of RSSPA across the entire ENCODE region. The positive regions in the pseudo ROC are derived from the known 5'ends (RefSeq) ± 500 bp of the first exon(and UTR). The negative regions are derived from intergenic regions as well as the inner-most introns of transcripts. While the true performances of the algorithms are not precisely determined in the pseudo ROC curves the estimation of their relative performance is accurate.

A pseudo receiver operating characteristic (ROC) curve method [39,40] has also been employed to characterize the performance of RSSPA. In the absence of a gold standard, for this analysis the positive regions are derived from the known 5' ends (RefSeq) ± 500 bp of the first exon(and UTR). The negative regions are derived from intergenic regions as well as the inner-most introns of transcripts provided their bounds do not overlap with the positive regions. Since RNA pol II occupancy sites, internal to transcripts and/or in intergenic space have been validated by qPCR the above definition of the negative regions might include true positive sites. Similarly, the definition of positive regions might include true negatives. Therefore, there is some degree of contamination in the delineation of the positive and negative regions, hence the pseudo nature of this analysis. The pseudo-ROC curves provide an accurate estimation of the relative performances of the various algorithms. Fig. 11 compares the performance of RSSPA versus binary segmentation via ROC curves; the x-axis corresponds to the FPR (1 – specificity) and the y-axis corresponds to sensitivity. The solid curves are representative of RNA pol II data derived from chromosome 1 as sampled by the ENCODE array. The performance of RSSPA (orange) has been contrasted with that of binary segmentation using pScore thresholds of 50 (blue), 40 (red) and 30 (magenta). At low FPR (≤ 0.1) a greater than 4x improvement in sensitivity is observed in the RSSPA over the latter. As expected, within the binary segmentation approaches, the sensitivity improves with increased pScore threshold; this comes at a price of increased false negative rate. The dotted curve is representative of RNA pol II data derived from all chromosomes sampled by the ENCODE array. Use of the partial area under the curve (pAUC) [41] metric yields a recovery of approximately 83.6 and 95.09 percent of *true *positive occupancy sites at 1 and 5 percent FPR respectively.

**Figure 12:**
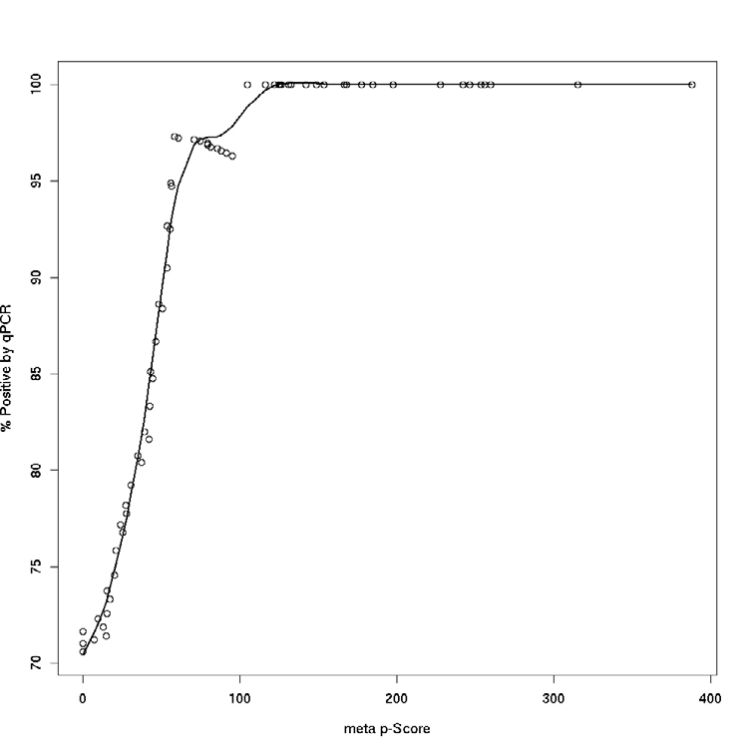
Validation of RSSPA using qPCR data – I. This plot shows the percentage of positive validation of HisH4 acetylation sites by qPCR (x-axis) as a function of the *meta *p-value, represented as pScore (y-axis). A random sampling of RSSPA-predicted sites comprising the entirety of the rank spectrum was validated by qPCR. The data shows that at a minimum *meta *pScore of ~56, 95% of the predicted sites were validated by qPCR.

**Figure 13:**
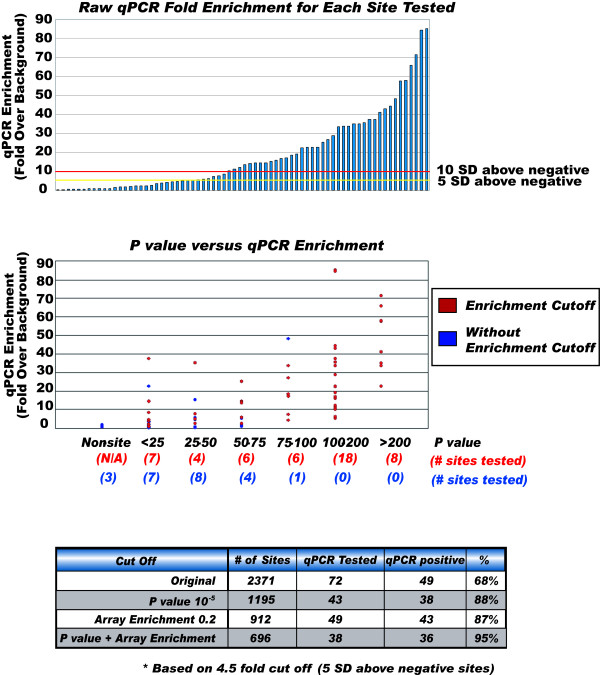
Validation of RSSPA using qPCR data – II. qPCR validation data is shown for HisH4 sites generated via RSSPA. (Top panel): Raw qPCR enrichment (fold over background) for each site tested. Two qPCR enrichment thresholds corresponding to 5 SD (yellow) and 10 SD (red) above negative sites are shown. The sites validated were selected at random from two distributions: (i) *meta *p-value (ii) signal enrichment. (Center panel): The qPCR enrichment of the sites validated has been binned against their *meta *p-value distribution. The results attest to a positive correlation between the two variables. Data-points in blue represent tested sites which are selected without any SE filter and the ones in red indicate sites selected following the application of a SE = ln(2) filter. The strong correlation between p-value and SE observed in the computational analysis is validated by qPCR. (Bottom panel): A summary of the sensitivity of RSSPA as obtained with different combination of p-value and signal enrichment filters employed in final segmentation.

Fig. 12, 13 summarizes the HisH4 qPCR validation data. A random sampling of n = 72 RSSPA-predicted sites comprising the entirety of the rank spectrum is validated by qPCR. Negative controls are designed from regions on the array which are not predicted as sites. These controls establish a baseline to ascertain whether a site's qPCR enrichment is positive and hence determine the sensitivity and specificity of the proposed algorithm. Fig. 12 illustrates the association of a qPCR based FPR with the statistical significance estimated by RSSPA. This in effect is a biological characterization of the performance of the algorithm. The plot represents the percentage of predicted acetylation sites that are validated by qPCR (y-axis) as a function of the *meta *p-value (y-axis). The qPCR based true positive rate is estimated by considering the number of validated sites as a fraction of the total number of sampled sites at a given pScore threshold. The range of the tested pScore is from 0–390. A pScore threshold of approximately 56 corresponds to a 95 percent FPR. Aside from providing a methodology to characterize the performance of the algorithm, this also enables an experimentalist to generate an initial ranked list of enrichment sites then further segment sites based on an experimentally derived pScore threshold. Fig. 13 (top panel) delves into the discussion of how true positives are determined based on qPCR validation. qPCR enrichment values for the 72 sites are shown (top panel) and thresholds are set based on 5σ (yellow) or 10σ (red) above the mean of the negative and non-sites(m = 3). Sites with enrichment above these thresholds are considered true positives. Fig. 13 (center panel), summarizes the data discussed in Fig 12, and further classifies sites into two groups based on whether they meet a 0.2 SE threshold (red) or do not (blue). The primary conclusions here are:

(i) There is a trend of positive correlation between p-value and qPCR enrichment, with R^2 ^= 0.45 (data not shown); the reduced concordance is partially attributable to the fact that the qPCR experiments are conducted using an un-amplified sample whereas the arrays are hybridized to an amplified sample.

(ii) With increasing p-value, there is a higher percentage of overlap with sites that pass the stipulated array enrichment threshold. While true positives are observed in the lower p-value bins, there is a higher degree of contamination due to auto-correlation artifacts, the majority of which are eliminated by the SE filter. Additionally, with the exception of a few outliers, the higher order p-value bins tend to have higher qPCR enrichments.

The primary conclusion therefore, is that in order to achieve a low percent FDR in site prediction, both p-value and SE thresholds need to be employed. A five percent FDR is optimal for most factors studied here (data not shown). The sensitivities (Fig 13: bottom panel) obtained subsequent to the segmentation of data using the filters: (a) *meta *p-value of 10^-5 ^(b) array signal enrichment of 0.2 (c) the composite of (a) and (b) are 88%, 87% and 95% respectively.

## Discussion

Positive probe thresholds coupled with the stringency of the maxgap and minrun control the degree of initial data fragmentation, affecting the sensitivity and specificity of the subsequent analyses. Increasing the stringency of the parameters introduces a potential bias towards false negatives and vice-versa. While a false negative bias is conservative, it obscures identification of sites with low enrichment profiles. Conversely, a false positive bias results in lower SNR. This compromise is partially dictated by the biological investigation at hand. In an exploratory mode, a false positive bias might be preferred. Alteration in any of these analysis strategies and parameters results in different, but overlapping transcription-regulation maps. The analysis goal is to strike a balance via co-optimization of sensitivity and specificity.

RSSPA does not incorporate explicit corrections for the following: (a) probe affinity; (b) auto-correlation. In the ideal model, the probe to site relationship should remain constant across replicates. Therefore cumulative probe affinity for a given site should be a constant across all replicates and have no impact on the assessment of inter-replicate rank consistency. In reality, the probe to site relationship varies across replicates; hence a correction factor for this covariate might improve the sensitivity of the analysis. Sites impacted by auto-correlation are inherently ranked lower and cannot be validated by qPCR. However, if the algorithm is used to both rank sites and segment them, based on λ and/or SE estimates, then specificity can be improved by modeling the underlying autocorrelation. Mechanisms for facilitating the estimation and/or de-convolution of autocorrelation have been discussed in the *methods *section. It should be emphasized that the sources of error in a ChIP assay are manifold. The primary ones are antibody specificity, fragmentation variance and amplification errors. Accurate estimation of the site span and enrichment requires a rigorous approach such as propagation of error, estimated at each stage of the ChIP on chip experimental procedure. Nonetheless, the proposed algorithm provides a high-sensitivity and high-specificity predictive mechanism to corroborate known elements, and catalog putative and novel elements of the regulatory network.

Replicate-statistics is a critical element of RSSPA. The experimental design must include at least two replicates and the number of replicates in the treatment and control samples must be balanced. The algorithm does not enforce a minimum correlation across replicates, although from the discussion of the covariates it should be clear that a lack of reproducibility across experiments will adversely affect the sensitivity and specificity of the outcome. Finally, disparity within the control experiments, to the extent they generate sites of spurious enrichment, can have adverse effects on the outcome. While the data normalization mitigates this significantly, a pre-filtering of the control data based on the outcome of least-squares linear fit can further improve the outcome. In summary, it is the reproducibility rather than the absolute number of replicates that has a stronger impact on the performance of RSSPA.

Since RSSPA is a non-parametric technique it is worthwhile to compare it with site prediction based upon the Hidden Markov Model (HMM). HMM is fundamentally suited to a problem of this nature, but its efficacy depends upon the appropriateness of the state transition matrix employed. HMM applications do not consider an explicit state duration density. They assume the fundamental state duration is exponential. For sequence specific factors such as p53, Sp1, this exponential model is appropriate, in most circumstances. For histone modification factors, however, variance in the binding footprints might actually require a Hidden semi-Markov Model approach, to prevent a significant false positive bias.

## Conclusion

RSSPA circumvents several sources of error common to parametric methods of ChIP on chip enrichment detection. It is based on a simple set of assumptions which have been validated by experiments, resulting in simplicity of implementation that allows users to choose whether initial estimates from the data are based upon metrics of statistical confidence (p-value) or signal enrichment. Independent of this initialization, the underlying multivariate optimization makes use of both metrics – this is where the power of the method lies. The requirement of replicate experiments should not be construed as a limitation, since prediction of regulatory targets based upon single data-points is a highly flawed approach due to the significant source of variance in the experiments themselves. By using a rank consistency approach across replicates, RSSPA actually utilizes the biological variance to associate statistical confidence to site prediction. The algorithm also allows flexibility of output type. The output can be a ranked list of predicted sites or a segmented list of sites following the application of a threshold. In summary, RSSPA is not microarray platform specific and does not require the presence of both PM and MM probes. The FDR associated with the predicted site-list provides correction for multiple hypothesis testing and enables comparison of results across microarray platforms.

## Authors' contributions

SG developed and implemented the algorithm; all code is written in R [42] version 2.0.1. NS and HAH was involved in ChIP sample generation and independent validation using qPCR and molecular biology approaches. TRG and KS were involved in sample and array data generation and overall guidance in the project. SG wrote the manuscript and all authors read and approved the final version of it.
